# Spatial Attention Effects of Disgusted and Fearful Faces

**DOI:** 10.1371/journal.pone.0101608

**Published:** 2014-07-01

**Authors:** Dandan Zhang, Yunzhe Liu, Chenglin Zhou, Yuming Chen, Yuejia Luo

**Affiliations:** 1 Institute of Affective and Social Neuroscience, Shenzhen University, Shenzhen, China; 2 State Key Laboratory of Cognitive Neuroscience and Learning, Beijing Normal University, Beijing, China; 3 School of Kinesiology, Shanghai University of Sport, Shanghai, China; University of Udine, Italy

## Abstract

Effective processing of threat-related stimuli is of significant evolutionary advantage. Given the intricate relationship between attention and the neural processing of threat-related emotions, this study manipulated attention allocation and emotional categories of threat-related stimuli as independent factors and investigated the time course of spatial-attention-modulated processing of disgusting and fearful stimuli. The participants were instructed to direct their attention either to the two vertical or to the two horizontal locations, where two faces and two houses would be presented. The task was to respond regarding the physical identity of the two stimuli at cued locations. Event-related potentials (ERP) evidences were found to support a two-stage model of attention-modulated processing of threat-related emotions. In the early processing stage, disgusted faces evoked larger P1 component at right occipital region despite the attention allocation while larger N170 component was elicited by fearful faces at right occipito-temporal region only when participants attended to houses. In the late processing stage, the amplitudes of the parietal P3 component enhanced for both disgusted and fearful facial expressions only when the attention was focused on faces. According to the results, we propose that the temporal dynamics of the emotion-by-attention interaction consist of two stages. The early stage is characterized by quick and specialized neural encoding of disgusting and fearful stimuli irrespective of voluntary attention allocation, indicating an automatic detection and perception of threat-related emotions. The late stage is represented by attention-gated separation between threat-related stimuli and neutral stimuli; the similar ERP pattern evoked by disgusted and fearful faces suggests a more generalized processing of threat-related emotions via top-down attentional modulation, based on which the defensive behavior in response to threat events is largely facilitated.

## Introduction

Rapid detection of impending danger is crucial for the interplay between humans and their environment. Our neural system has evolved to allow the expedient perception of potentially aversive stimuli [1]. In particular, humans always grant priority of attention allocation to threat-related stimuli; compared with non-threat events, humans disengage the fixation of attention more difficultly and less frequently from potentially dangerous events [2]. However, previous studies also suggested that the brain sometimes responses to threat automatically at the pre-attentive level [3], [4]. Furthermore, attention may not be mandatory for the neural processing of all the threat-related information [1], [5], [6]. For example, both adults and young infants can response to threat involuntarily, without the focus of attention or even without the awareness of the stimulus occurrence [1], [7], [8].

Given the intricate relationship between emotion processing and attention, there is a need to manipulate attention allocation and emotional characteristics of stimuli (e.g., threat-related *vs*. non-threat-related; high-arousal *vs*. low-arousal) as independent factors and to investigate the interaction between them. However, most previous studies did not unambiguously discriminate between the focus of attention and emotion processing itself, thus failed to demonstrate whether the privileged neural processing of threat-related information is independent of attention modulation [1], [4], [6]–[8]. Regarding the few studies that successfully distinguished the effects of attention and threat-related emotions, the results were inconsistent. For example, one functional magnetic resonance imaging (fMRI) study found that the blood-oxygen-level dependent (BOLD) signal of amygdala was larger in response to fearful faces as compared to neutral ones [9]. The authors also found that this emotion effect was not modulated by attention, suggesting that fearful stimuli may be detected pre-attentively [9]. Conversely, another fMRI study indicated that the attention influenced the amygdala function; compared with the unattended condition, the amygdala showed larger activity in the attend-to-fearful-face condition [10]. The discrepancy in these two studies may be due to the intrinsic limitation of fMRI technique: the BOLD signal varies slowly as compared to the rapidly-changed neuroelectrical activity, which may prevent the fMRI device from capturing the transient fluctuations of neural characteristics in these studies [11].

Meanwhile, although threatening events are typically associated with heightened neural responses [8], [12], the model of threat-related processing is usually oversimplified with almost exclusive focus on the emotion of fear; other threat-related emotions have been overlooked in most of the previous literatures [13]. In the present study, we investigated and compared two subtypes of threat-related emotions, namely, fear and disgust. These two emotions represent different biological systems—the “self protection system” [14] and the “disease avoidance system” [15], respectively. Previous researches have demonstrated that fearful and disgusting emotions could induce divergent physiological responses and cognitive processes, i.e., disgust tends to activate parasympathetic system and suppresses action while fear stimulates sympathetic pathways and prompts fight or flight [16]; disgust provokes instant sensory rejection, whereas fear quickly orients attention so as to ensure sensory acquisition [17]. Vermeulen, Godefroid, & Mermillod employed an attentional blink task (with emotional faces as primes) and found that compared with the neutral faces, disgusted faces were associated with reduced attentional blinks [18]. One recent event-related potential (ERP) study asked participants to search the horizontal bar among seven vertical bars with fearful, disgusting or neutral affective pictures as visual background, which found a rapid discrimination between the two threat-related emotions as early as 96 ms after stimulus onset, represented by larger occipital P1 amplitudes in fearful condition and smaller P1 amplitudes in disgusting condition, compared with those in neutral condition [19]. However, while disgust is frequently considered as a warning signal for biological/psychological contamination and usually results in avoidant behavior [15], a few studies indicated that disgusting stimuli sometimes capture attention even faster than fearful stimuli [20]. For example, it is found in a masked presentation task that participants responded faster to disgusting words than to fearful or neutral words [21]. It is believed that the quick, early attention effects on different threat-related emotions could be further disclosed using the ERP technique, which has a high time resolution and could follow the neural dynamics timely.

Relevant studies have also suggested that after the early specialized processing, as described above, the threat-related emotions are further analyzed via a relatively general procedure with top-down modulation so as to facilitate subsequent defensive behavior; and that the attentional resources are necessary and essential at this stage. For example, one ERP study found that after an early P1 discrimination (peaked at 115 ms post-stimulus) between fearful and disgusting pictures, late ERP components within the time window of 388–425 ms converged between the two threat-related emotions [22]. In another ERP study, participants were presented with a rapid and continuous stream of high- and low-arousing affective pictures; the researchers found that high-arousing pictures evoked larger N2 amplitudes (∼200 to 350 ms after stimulus onset) than low-arousing pictures both in attended and unattended conditions; in contrast, the P3 amplitudes (∼400 to 600 ms) were markedly enhanced in high-arousing condition compared with low-arousing condition only when participants paid attention to the affective pictures [2]. Thus, unlike the early stage of the neural processing of threat-related emotions, the later stage may demand sufficient attention focused on target stimuli.

The present study employed ERPs to investigate whether the neural processing of fearful and disgusting stimuli is independent of attentional modulation. We manipulated spatial attention (i.e., stimuli appeared at attended or unattended locations) and emotional categories of presented stimuli as independent factors and examined the time course of the emotion-by-attention interaction. We hypothesized that fear and disgust may have distinct encoding patterns at early stage of attention-modulated processing, followed by a late, more generalized processing of threat-related information that may be strongly influenced by attention. Previous ERP studies have indicated that two early ERP components, namely the occipital P1 and the occipito-temporal N170, are sensitive to both attention and emotion effects [1], [23]–[25]. Besides P1 and N170, the N2pc has recently been proposed to be an effective biomarker of attention shift or selection [26]. For example, when the facial configuration contained both eyebrows and eyes, threatening angry targets showed a more pronounced occipital N2pc between 200 and 300 ms than friendly facial targets, which indicated that the advantage of rapid prioritized attention to facial threat is not driven by low-level visual features [27]. In addition, the centro-parietal P3 is typically found to reflect the neural process of selective spatial attention and serves as a measure of top-down modulation [28]. Therefore, it is expected that early ERP components such as P1 and N170 would show different patterns between emotion subtypes of fear and disgust, and that the amplitudes of later ERP components (e.g. P3) may be enhanced in both fearful and disgusting conditions, but only when participants paid attention to emotional stimuli.

## Methods

### Participants

Thirty-one healthy subjects (15 females; age range  = 21 to 27 years) were recruited from Beijing Normal University in China as paid participants. All participants were right-handed and had normal or corrected-to-normal vision. They gave their written informed consent prior to the experiment. The individuals whose photographs are shown in this manuscript have given written informed consent (as outlined in PLOS consent form) to publish their photographs. The experimental protocol was approved by the local ethics committee (Beijing Normal University).

### Stimuli

Faces were black and white photographs selected from the native Chinese Facial Affective Picture System (CFAPS) [29], with equal number of face pictures between males and females. A total of 60 faces (20 disgusted, 20 fearful, and 20 neutral faces) were used. Each picture had been assessed for its valence and arousal on a 9-point scale with a large sample of Chinese participants in a previous survey. The ANOVA performed on the average scores showed that the two categories of negative faces did not differ significantly in emotional valence (*F*(2,38) = 172, *p*<.001, 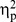  = .900; mean ± standard deviation (SD): disgust  = 3.24±0.33, fear  = 3.06±0.38, neutral  = 4.81±0.24; disgust *vs*. fear: *p* = .433) or arousal (*F*(2,38) = 1.54, *p* = .227, 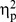  = .075; disgust  = 5.69±0.45, fear  = 5.78±0.52, neutral  = 5.50±0.35; disgust *vs*. fear: *p* = 1.000) while their valence ratings significantly differed from neutral faces (*p*s<.001). Of note, to prevent our results from being contaminated by the arousal across three emotional conditions, the 20 neutral faces were selected as with a relatively high arousal from a total of 422 neutral faces in the CFAPS (valence  = 4.29±0.52; arousal  = 3.84±0.69 of the 422 neutral faces). A total of 60 pictures of front-view houses were selected from internet. All stimuli were presented with the same contrast and brightness on the black background (3.0°×3.5° visual angle).

### Procedure

The experimental procedure was similar to those employed in previous fMRI [9], [30], [31] and ERP studies [32]. Participants were seated in a dimly lit and sound-attenuated room. Stimuli were presented on a LCD monitor at a viewing distance of 100 cm. The experiment consisted of six blocks, each containing 64 trials.

**Figure 1:**
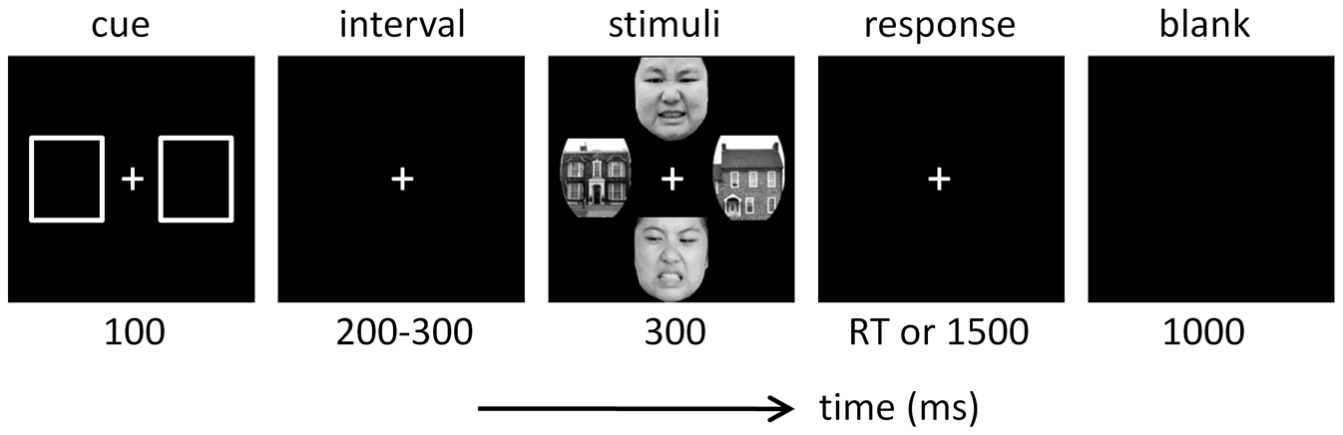
Illustration of one experimental trial in this study.

Stimulus display and behavioral data acquisition were conducted using E-Prime 1.2 (Psychology Software Tools, Inc., Pittsburgh, PA). During the experiment, participants were required to always fix their eyes on the white cross in the center of the screen. As shown in Figure 1, each trial started with a 100-ms cue that consisted of two white rectangles (3.0°×3.5° visual angle). The cue instructed subjects to direct their attention either to the two vertical or to the two horizontal locations; the stimulus pair at uncued locations should be ignored. After the cue, an interval was presented with the duration of 200 to 300 ms. Then two faces and two houses were presented for 300 ms. The two faces in each trial have the same emotion category and the same gender, which were selected randomly from one of the three facial expression categories and from one of the two genders. After the presentation of face/house stimulus array, subjects were required to respond as quickly and accurately as possible regarding the physical identity of the two stimuli at cued locations, with a “yes” key for an identical pair and a “no” key for a different pair. The response screen would not disappear until a button press or until 1500 ms elapsed. The inter-trial interval was 1000 ms. Participants were instructed to press the “F” and “J” buttons on the computer keyboard with their left and right index fingers. The assignment of keys to “yes” and “no” responses was counterbalanced across participants. In each block, the location (vertical *vs*. horizontal) of face and house pairs varied randomly across trials. The vertical and horizontal positions were equally likely cued or uncued.

### EEG recording and ERP analysis

Brain electrical activity was recorded referentially against left mastoid and off-line re-referenced to the average of the left and right mastoids, by a 64-channel amplifier with a sampling frequency of 250 Hz (NeuroScan Inc., Herndon, USA). Besides electrooculogram electrodes, a 62-channel electroencephalography (EEG) data were collected with electrode impedances kept below 5 kΩ. Both vertical and horizontal ocular artifacts were removed from the EEG data using a regression procedure implemented in Neuroscan software (Scan 4.3). In particular, two electrooculogram templates (one for vertical and one for horizontal eye movements) were calculated from the EEG data. Then the software removed the ocular artifacts by performing two regression procedures.

The data analysis and result display in this study were performed using Matlab R2011a (MathWorks, Natick, USA). The recorded EEG data were filtered with a 0.01–30 Hz finite impulse response filter with zero phase distortion. Filtered data were segmented beginning 200 ms prior to the onset of face/house stimulus array and lasting for 1200 ms. All epochs were baseline-corrected with respect to the mean voltage over the 200 ms preceding the onset of the face/house stimulus array, followed by averaging in association with experimental conditions.

In the present study, we focused on the ERPs elicited by disgusted, fearful, and neutral facial expressions and in attend-to-face and attend-to-house conditions. The individual average ERPs of the 31 subjects were computed based on behaviorally correct trials, thus leading to 9.92±5.84 trials (mean ± SD) being excluded from the data per condition per subject (the minimum number of accepted trials per condition in the individual average ERP was 39). The data were derived from all electrodes, but only the electrodes at which the components reached their peak values were entered into statistical analysis. We analyzed the potentials of occipital P1, occipito-temporal N170, and parietal P3 components across different sets of electrodes according to grand-mean ERP topographies. Time windows for mean amplitude calculation were centered at the peak latencies of ERP components in grand-mean waveforms, with a shorter window length for early components and a longer length for late component. The mean amplitudes of P1 were calculated at O1 and O2 within the time window of 100–130 ms [19], [22]. The mean amplitudes of N170 were calculated at P7 and P8 within the time window of 170–200 ms [1], [23]. The mean amplitudes of P3 were calculated at CPz and Pz within the time window of 520–680 ms [2], [33].

### Statistics

Statistical analyses were performed using SPSS Statistics 20.0 (IBM, Somers, USA). Descriptive data were presented as mean ± SD. The significance level was set at 0.05. Two-way repeated-measures ANOVAs were performed on measurements of accuracy rate (ACC), reaction time (RT), and the P3 amplitude, with emotion (disgust, fear, and neutral) and attention (attend to faces and attend to houses) as the two within-subject factors. Three-way repeated measures ANOVAs on the amplitudes of P1 and N170 components were conducted with emotion, attention, and hemisphere (left and right) as within-subject factors. Greenhouse-Geisser correction for ANOVA tests was used whenever appropriate. Post-hoc testing of significant main effects was conducted using Bonferroni method. Significant interactions were analyzed using simple effects model. Partial eta-squared (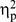) was reported to demonstrate the effect size in ANOVA tests, where 0.05 represents a small effect, 0.10 indicates a medium effect, and 0.20 represents a large effect. For the sake of brevity, effects that did not reach significance have been omitted.

## Results

### Behaviors

**ACC**. The main effect of emotion was significant (*F*(2,60) = 25.1; *p*<.001; 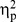  = .456). The ACC in neutral condition (0.812±0.107) was smaller than that in disgusting (0.862±0.081; *p*<.001) and fearful conditions (0.861±0.075; *p*<.001).

The main effect of attention was significant (*F*(1,30) = 32.4; *p*<.001; 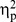  = .519). The ACC in attend-to-face trials (0.812±0.102) was smaller than that in attend-to-house trials (0.878±0.065).

The interaction effect of emotion by attention was significant (*F*(2,60) = 27.5; *p*<.001; 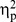  = .478). Simple effect analysis showed that the emotion effect was significant in attend-to-face condition (*F*(2,60) = 47.7; *p*<.001; disgust  = 0.843±0.089; fear  = 0.847±0.083; neutral  = 0.747±0.101) while there was no significant emotion effect in attend-to-house condition (*F*(2,60)<1; disgust  = 0.880±0.069; fear  = 0.876±0.063; neutral  = 0.878±0.065).

**RT**. The main effect of emotion was significant (*F*(2,60) = 6.07; *p* = .004; 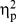  = .168). The RT in neutral condition (773±89.7 ms) was longer than that in disgusting condition (741±87.2 ms; *p* = .006) while the neutral and fearful (761±91.9 ms) conditions showed no significant difference (*p* = .123).

The main effect of attention was significant (*F*(1,30) = 4.76; *p* = .037; 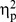  = .137). The RT in attend-to-face trials (765±96.5 ms) was larger than that in attend-to-house trials (752±83.2 ms).

The interaction effect of emotion by attention was significant (*F*(2,60) = 10.5; *p*<.001; 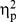  = .259). Simple effect analysis showed that the emotion effect was significant in attend-to-face condition (*F*(2,60) = 10.2; *p*<.001; disgust  = 741±91.7 ms; fear  = 764±98.3 ms; neutral  = 789±96.3 ms) while there was no significant emotion effect in attend-to-house condition (*F*(2,60) = 2.07; *p* = .135; disgust  = 741±84.0 ms; fear  = 758±86.4 ms; neutral  = 757±80.8 ms).

### ERPs

**P1.** The main effect of emotion was significant (*F*(2,60) = 5.14; *p* = .009; 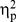  = .146). The P1 amplitude in response to disgusted faces (1.75±1.45 µV) was larger than that in response to fearful (1.40±1.79 µV; *p* = .015) and neutral faces (1.40±1.68 µV; *p* = .017).

The main effect of attention was significant (*F*(1,30) = 9.97; *p* = .004; 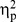  = .249). The P1 amplitude was larger when participants paid attention to faces (1.66±1.68 µV) than to houses (1.37±1.61 µV).

**Figure 2:**
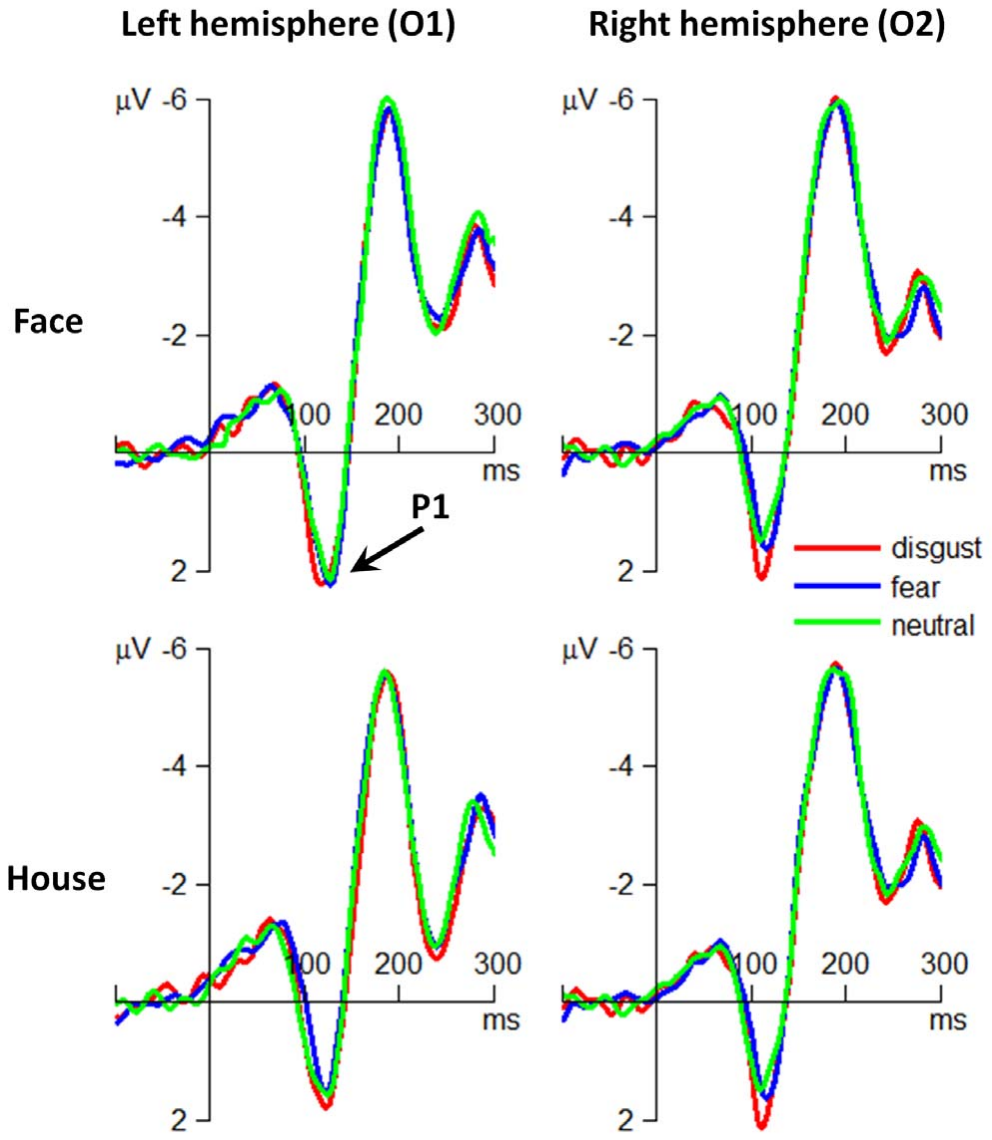
The grand-mean ERP waveforms at the occipital electrode sites of O1 and O2.

The interaction effect of emotion by hemisphere was significant (*F*(2,60) = 3.45; *p* = .038; 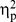  = .103) (Figure 2). Simple effect analysis indicated that the emotion effect on P1 was significant at the right hemisphere (*F*(2,60) = 9.11; *p*<.001); the disgusted faces (1.94±1.23 µV) elicited larger P1 amplitudes than did fearful (1.29±1.56 µV) and neutral faces (1.27±1.49 µV). However, this emotion effect was not significant at the left hemisphere (*F*(2,60)<1).

**N170.** The main effect of attention was significant (*F*(1,30) = 44.8; *p*<.001; 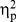  = .599). The N170 amplitude was larger in attend-to-face condition (−6.27±4.39 µV) compared with that in attend-to-house condition (−4.66±3.62 µV).

The main effect of hemisphere was significant (*F*(1,30) = 19.5; *p*<.001; 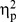  = .394). The N170 amplitude in the left hemisphere was smaller (−3.70±2.94 µV) than that in the right hemisphere (−7.23±4.33 µV).

The interaction effect of emotion by hemisphere was significant (*F*(2,60) = 6.05; *p* = .004; 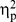  = .168). Simple effect analysis indicated that the emotion effect on N170 was significant at the right hemisphere (*F*(2,60) = 9.22; *p*<.001); the N170 elicited by fearful faces (−7.72±4.43 µV) was larger than that elicited by disgusted (−7.04±4.31 µV) and neutral faces (−6.93±4.28 µV). However, this emotion effect was not significant at the left hemisphere (*F*(2,60)<1).

The interaction effect of attention by hemisphere was significant (*F*(1,30) = 21.6; *p*<.001; 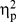  = .419). The attention effect was more significant at the right hemisphere (*F*(1,30) = 48.1; *p*<.001) than at the left hemisphere (*F*(1,30) = 11.3; *p* = .002). The N170 in attend-to-face condition (left  = −4.10±3.10 µV; right  = −8.45±4.42 µV) was larger than that in attend-to-house condition (left  = −3.31±2.73 µV; right  = −6.01±3.90 µV).

The interaction effect of emotion by attention was significant (*F*(2,60) = 4.08; *p* = .032; 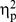  = .120). The emotion effect was significant when participants attended to houses (*F*(2,60) = 5.61; *p* = .006); the N170 elicited by fearful faces (−5.08±3.83 µV) was larger than that elicited by disgusted (−4.45±3.52 µV) and neutral faces (−4.44±3.52 µV). However, this emotion effect was not significant when participants attended to faces (*F*(2,60)<1).

**Figure 3:**
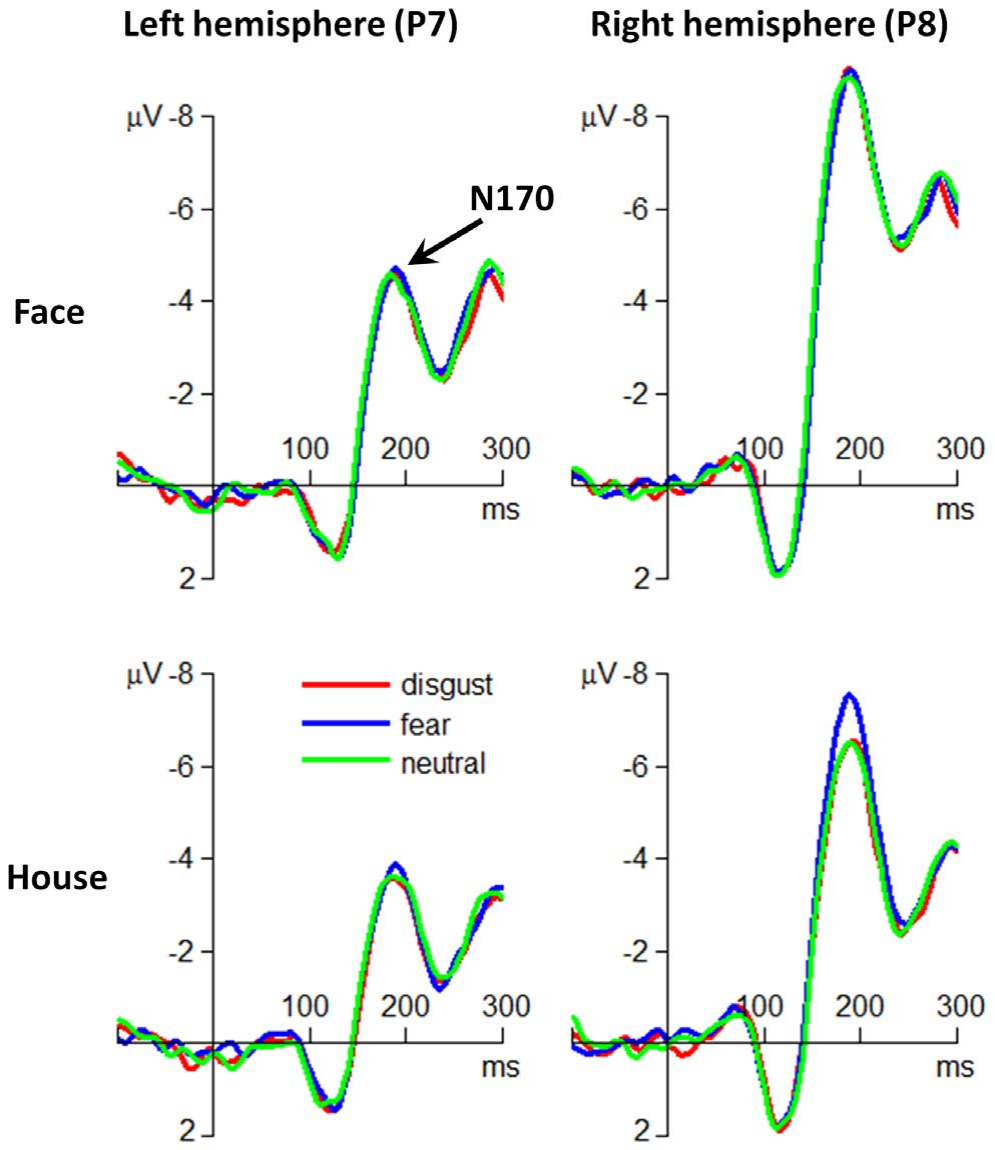
The grand-mean ERP waveforms at the occipito-temporal electrode sites of P7 and P8.

The interaction effect of emotion by attention by hemisphere was significant (*F*(2,60) = 3.42; *p* = .043; 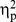  = .102) (Figure 3). Simple simple effect analysis indicated that the emotion effect on N170 was significant only at the right hemisphere and only in attend-to-house condition (*F*(2,60) = 18.4; *p*<.001); the N170 elicited by fearful faces (−6.86±4.12 µV) was larger than that elicited by disgusted (−5.60±3.78 µV) and neutral faces (−5.57±3.75 µV).

**Figure 4:**
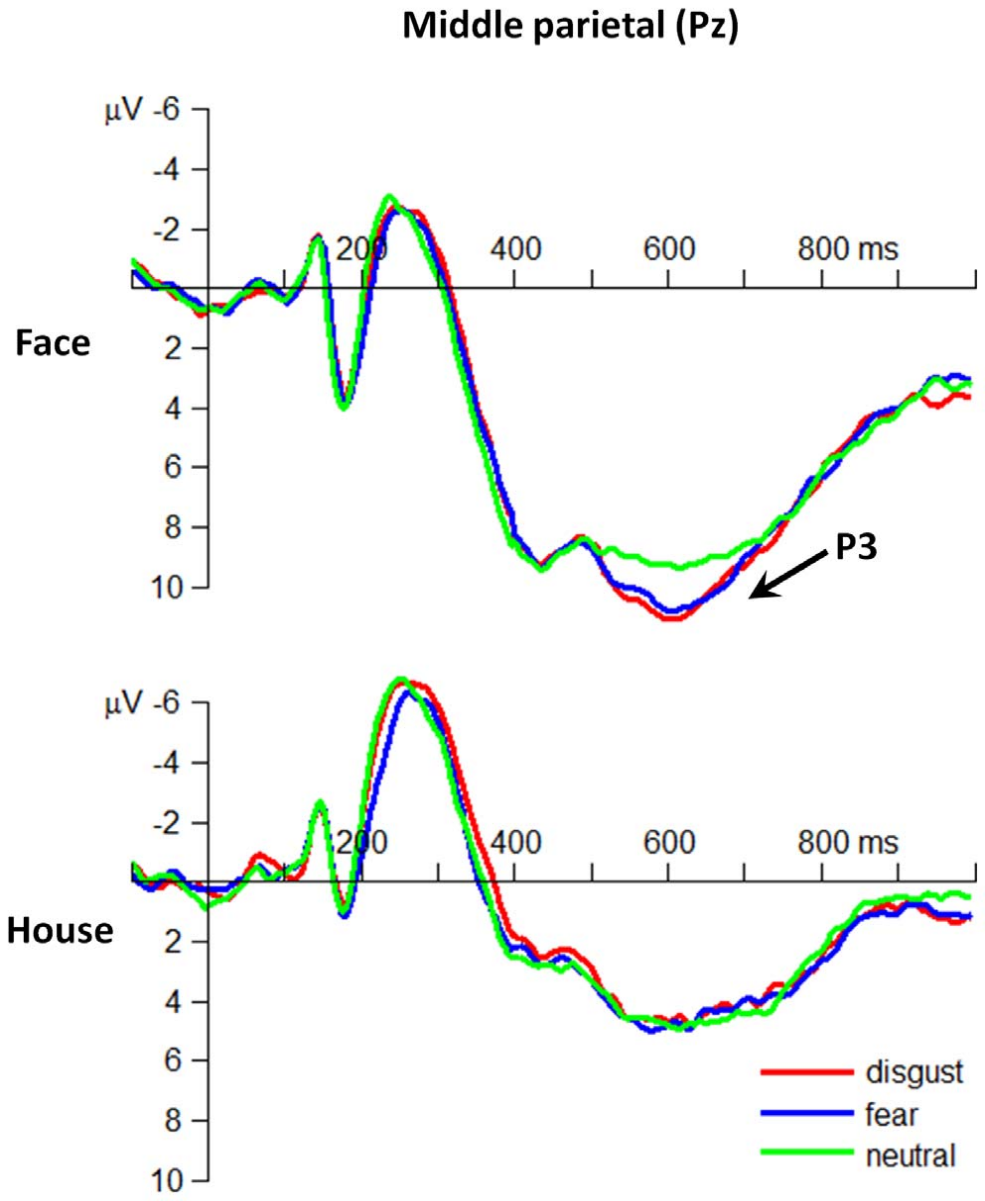
The grand-mean ERP waveforms at the middle parietal electrode site of Pz.

**P3.** The main effect of emotion was significant (*F*(2,60) = 6.22; *p* = .004; 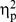  = .172) (Figure 4). The P3 amplitudes in response to disgusted (7.91±3.41 µV; *p* = .025) and fearful faces (7.69±4.06 µV; *p* = .028) were larger than those in response to neutral faces (6.63±4.16 µV).

The main effect of attention was significant (*F*(1,30) = 210; *p*<.001; 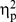  = .875). The P3 amplitude was larger when participants attended to faces (9.89±3.32 µV) than to houses (4.92±2.70 µV).

**Figure 5:**
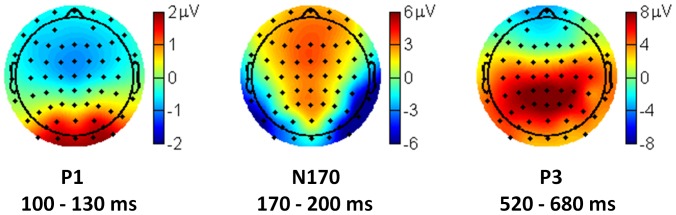
The grand-mean topographies of the P1, the N170, and the P3 components.

The grand-mean topographies of the ERP components of P1, N170, and P3 are shown in Figure 5.

## Discussion

This study investigated the time course of the interaction effects between spatial attention and the two subtypes of threat-related emotions (fear vs. disgust). At the behavioral level, it was observed that fearful and disgusting stimuli resulted in higher ACC and shorter RT measures as compared to neutral stimuli, and that this phenomenon was only significant when participants attended to faces. At the electrophysiological level, we found neural evidences supporting a two-stage model of attention-modulated processing of threat-related emotions. The early stage was represented by the P1 and the N170 components that distinguished disgusting and fearful stimuli from neutral ones, respectively. In particular, the P1 component showed larger amplitudes in disgusting than in fearful and neutral conditions, irrespective of attention allocation. Meanwhile, the N170 component displayed larger amplitudes in fearful than in neutral and disgusting conditions when participants attended to houses. The late processing stage was represented by the P3 component, which was enhanced in response to both disgusted and fearful facial expressions when participants attended to faces.

The most novel finding of the present study is the neural evidence for early discrimination of disgusted faces, as evidenced by the larger P1 amplitude in disgusting condition compared with that in fearful and neutral conditions. The occipital P1 component has been proved to be sensitive to early emotional modulation in visual perception [24], [25], [34]. In this study, the enhanced P1 amplitude in response to disgusted faces was robust in both attended and unattended conditions, suggesting that disgusted faces can be detected without focus of attention at the early stage of emotion-related processing. Of note, the larger P1 evoked by disgusted faces is not necessarily contradictory to previous studies suggesting that disgusting stimuli suppress subsequent cognitive processing. For example, Krusemark and Li [19] observed in a visual searching task that the P1 amplitude following disgusting picture presentation was smaller than following fearful and neutral picture presentations. While Krusemark and Li [19] mainly investigated the influence of emotional stimuli on the subsequent cognitive processing (i.e., visual searching task), this study focused on the direct influence of the presented threat-related stimuli on the current task. Our finding of the early P1 separation between disgusting and neutral/fearful stimuli is consistent with previous behavioral evidences that suggested a neutral processing bias to disgusting information [20], [21]. For example, people had more interference when responding to disgusting compared to fearful and neutral words in the Stroop color-naming task [20]; participants responded faster to disgusting words in the masked presentation task, compared with fearful and neutral conditions [21]. These converging behavioral and ERP evidences suggest a neutral bias to disgusting events/stimuli at early stage of emotion-related processing, which helps humans avoid potential contaminants timely and with a high success rate [21].

The early stage of attention-modulated emotion processing was also represented by larger occipito-temporal N170 amplitudes in fearful than in neutral and disgusting conditions. The N170 component is typically assumed to reflect structural encoding of faces and shows larger amplitudes for faces than other non-face objects [35], [36]. More recent findings have suggested that the N170 is also modulated by emotional faces, with larger amplitudes in response to fearful than neutral faces [9], [12], [23], [25], [37]. For example, employing a similar paradigm with in this study, Vuilleumier et al. [9] found that the right fusiform activity was influenced by emotional facial expressions, with a greater response to fearful than to neutral faces. However, the current finding seems to contradict the result obtained by Holmes et al. [5], who employed a very similar paradigm and found that the N170 component was unaffected by emotional facial expressions. The discrepancy between the present study and the Holmes' study may be due to the different tasks of participants (responses were required in a few *vs*. all trials in Holmes' and the present study, respectively) and/or different sets of electrode sites selected in the N170 analysis (T5 and T6 in Holmes' and P7 and P8 in this study). One of the most interesting results given by our data is the significant interaction between emotion and attention: the N170 showed larger amplitudes in fearful condition as compared to disgusting and neutral conditions only when participants attended to houses, i.e., when their attention was not focused on faces. It is known that fearful expressions are often associated with potential danger in the environment of which the source is usually undetermined [30]. It is of evolutionary advantage for fearful stimuli being processed rapidly, even when they occur in the periphery of the visual field [8], [13], [14]. Therefore, it is very likely that the attention capture effect of fearful faces is more prominent and easier to observe when participants pay their attention to emotion-irrelevant objects, such as the houses in this study [30]. In line with this interpretation, one previous fMRI research has found that amygdala showed larger activity in response to fearful faces, as compared to neutral and angry faces; and that this effect was only significant when the faces were out of attention [30].

The ERP data in the early processing stage of threat-related emotions indicated that the P1 component was able to separate disgust from fear and neutral in the attend-to-face condition. More importantly, we found that the discriminative index of disgusting stimuli (the P1) and fearful stimuli (the N170) were both valid in the unattended condition, suggesting that the early stage of threat-related processing may work automatically without specific attention allocation. In contrast, it was found that the later stage of emotion perception and interpretation was characterized as an attention-gated procedure, which was reflected by pronounced P3 amplitudes in fearful and disgusting conditions only when participants attended to faces. It has been proved that the amplitudes of the centro-parietal P3 component usually increase when the attention is allocated to target stimuli, as compared to unattended condition [38]. Therefore, it is believed that the P3 effect observed in this study indicated a voluntary attention modulation that occurred at the late stage of threat-related emotion processing [38], [39]. In addition, previous studies have also found an arousal effect of the P3 component, i.e., the P3 shows larger amplitudes in response to high-arousal stimuli compared with low-arousal or neutral stimuli [40], [41]. By matching the arousal level of all the three categories of facial expressions (fear, disgust and neutral), the current finding of enhanced P3 amplitudes in disgusting and fearful conditions was highly unlikely to be attributed to the difference of arousal, but rather indicated the attention-modulation effect on threat-related emotion processing. Given the potential relationship between the P3 component and the top-down modulation [42]–[44], we further suggest that the late stage of threat-related processing may reflect a procedure of voluntary allocation of attention to biologically important events, with potential neural substrates located in the dorsal fronto-parietal pathways[40], [45].

Some readers may note that the attend-to-face trials were associated with poorer performances (i.e., lower accuracy and longer reaction time) in this study. This result pattern may be due to the attention capture effect of facial expressions. It has been widely recognized that facial expressions are salient social signals, which tend to attract and hold attention more intensively as compared to other objects such as houses [46], [47]. In the current study, when participants attended to faces, their attention was held by the emotion of faces, resulting in less attentional resources for the discrimination task. Therefore, attend-to-face condition had poorer behavioral performances as compared to attend-to-house condition. Furthermore, this attention capture effect of facial expressions may be more significant when the faces are threat-related [48], [49]. Our finding is consistent with previous reports in the literature. For example, Vuilleumier et al. [9] found in a similar experiment that subjects made more errors in attend-to-face condition as compared to attend-to-house condition.

## Conclusion

To sum up, the current study investigated the time course of spatial-attention-modulated processes of disgusting and fearful stimuli. It is proposed that the temporal dynamics of this procedure consist of two stages. The early stage is characterized by quick and specialized neural encoding of disgusting and fearful stimuli irrespective of voluntary attention allocation, indicating an automatic detection and perception of threat-related emotions. The late stage is represented by attention-gated separation between threat-related stimuli and neutral stimuli; the similar ERP pattern evoked by disgusted and fearful faces suggests a more general process of threat-related emotions via top-down attentional modulation, based on which the defensive behavior in response to threat events is largely facilitated. Altogether, the current study reveals a systematic progression of the relationship between spatial attention and threat-related emotion processing, highlighting the adaptability of the human defense system which always optimizes its function to deal with diverse dangers in the environment.
