# Correction: Spatial Attention Effects of Disgusted and Fearful Faces

**DOI:** 10.1371/journal.pone.0107848

**Published:** 2014-09-04

**Authors:** The PLOS ONE Staff

In the Funding section, there is a missing grant number for the National Natural Science Foundation of China. Please refer to the complete funding statement below:

This study was funded by the National Basic Research Program of China (973 Program, 2014CB744600) and the National Natural Science Foundation of China (31300867, 31171004). The funders had no role in study design, data collection and analysis, decision to publish, or preparation of the manuscript.
